# Climate concern, pro-environmental behaviours and use of e-cigarettes in the European Union

**DOI:** 10.1093/eurpub/ckag114

**Published:** 2026-06-25

**Authors:** Charlotte Xin Li, Siyuan Fan, Ariadna Feliu, Anthony A Laverty, Filippos T Filippidis

**Affiliations:** Department of Primary Care and Public Health, School of Public Health, Imperial College London, London, United Kingdom; Department of Primary Care and Public Health, School of Public Health, Imperial College London, London, United Kingdom; Department of Primary Care and Public Health, School of Public Health, Imperial College London, London, United Kingdom; CIBER en Enfermedades Respiratorias (CIBERES), Madrid, Spain; Department of Primary Care and Public Health, School of Public Health, Imperial College London, London, United Kingdom; Department of Primary Care and Public Health, School of Public Health, Imperial College London, London, United Kingdom

## Abstract

This study examined whether broader environmental orientations are associated with current e-cigarette use and disposable e-cigarette use in the European Union (EU). We analysed data from Special Eurobarometer 99.3 (2023) across 27 EU Member States (*n* = 26,353), measuring environmental orientations via climate concern and pro-environmental behaviours. Climate concern was associated with lower likelihood of e-cigarette use (Prevalence Ratio = 0.84, 95% CI, 0.72–0.99); pro-environmental behaviour was associated with a lower likelihood of disposable e-cigarette use (0.72,0.53–0.99). Both estimates were attenuated in sensitivity analyses; the latter relied on a small subgroup. Findings should be considered exploratory. Further research using product-specific environmental measures is warranted.

## Introduction

E-cigarettes have become increasingly popular in the European Union (EU), particularly among young people [1]. Beyond their potential direct adverse health effects, e-cigarettes also pose environmental risks. Their components—including plastic casings, batteries, capsules, and atomisers—contribute to electronic waste, which may contain hazardous, toxic, and non-biodegradable materials, as well as metals such as cadmium, nickel and lead [2]. These concerns are particularly relevant for disposable e-cigarettes, which are designed for single use and are therefore discarded rapidly and in large quantities. Furthermore, e-liquids and aerosol emissions may release harmful substances into the environment [3].

Climate concern is widespread across the EU, with 60% of citizens willing to pay more for sustainable products [4]. This may be particularly relevant for younger people, a key population for e-cigarette use, who are also more likely to report environmentally conscious attitudes and behaviours [5]. Despite increasing recognition of the dual health and environmental impacts of e-cigarettes, previous studies in EU populations have mainly focused on health-related perceptions associated with e-cigarette use, such as perceived harmfulness and perceived usefulness for smoking cessation [6, 7]. We therefore examined whether broader environmental orientations, including climate concern and pro-environmental behaviours, were associated with e-cigarette use in the EU.

## Methods

We conducted a cross-sectional analysis of the most recent tobacco-related Eurobarometer wave (May–June 2023) across the 27 EU Member States (MS) (*n* = 26 353). The survey used a multistage sampling design to obtain nationally representative samples. Primary sampling units were selected within each MS according to population distribution, followed by household selection using standard random-route methods. One respondent aged ≥15 years from each household was interviewed face-to-face [1]. Data were weighted by age, gender, and area of residence.

Outcome variables were current e-cigarette use and current disposable e-cigarette use. Current e-cigarette use was assessed by asking: ‘Thinking about e-cigarettes, which of the following applies to you?’ Response options included: ‘You currently use it’, ‘You used to use it but you have stopped’, ‘You have tried it only once or twice’, ‘You have never used it’, and ‘Don’t know’. Respondents selecting ‘You currently use it’ were classified as current users, while all other responses were classified as non-current users; ‘Don’t know’ responses were treated as missing.

The disposable e-cigarette outcome was nested within current e-cigarette use. Current e-cigarette users were asked about the frequency of disposable device use. Responses of ‘Every day’, ‘Every week’, ‘Every month’, or ‘Less than monthly’ were classified as current disposable e-cigarette use; all other responses (‘You used to use it regularly but you have stopped’, ‘You have tried it only once or twice’, ‘Never’), as well as non-current e-cigarette use were classified as non-current disposable e-cigarette use, with ‘Refusal’ treated as missing.

Exposure variables included four measures. Climate concern was assessed using the question: ‘How serious a problem do you think climate change is at this moment?’ (scale 1–10) and we dichotomized it into very serious (scores 9–10) versus less than very serious (scores 1–8). This threshold was specified *a priori* to identify respondents reporting the highest levels of climate concern while maintaining a reasonably balanced distribution between exposure groups. Pro-environmental behaviours were assessed based on actions taken in the past 6 months, including (i) reducing and separating waste for recycling and (ii) reducing the use of disposable items, both coded as binary variables (yes/no). These measures were selected from the available Eurobarometer environmental behaviour items because they were considered most relevant to e-cigarette and disposable product use. A fourth binary variable captured engagement in at least one of these two behaviours to reflect broader engagement in pro-environmental practices.

Covariates included self-reported gender (man/woman), age (15–24/25–39/40–54/≥55 years), difficulty paying bills over the past 12 months (almost never/never versus from time to time/most of the time), area of residence (rural/urban), age at completion of full-time education (0–15/16–19/≥20 years/still studying), living with children (no/yes), and political affiliation (left/centre/right/don’t know or did not respond).

We applied official Eurobarometer survey weights to estimate the prevalence of exposure and outcome variables. Multilevel Poisson regression models were used to examine the associations of climate concern and pro-environmental behaviours with current e-cigarette and disposable e-cigarette use, adjusting for the covariates described above. Regression models were unweighted because key variables used in the Eurobarometer weighting procedure were included as covariates, and previous methodological research has suggested that unweighted estimates may be preferred when sampling weights are a function of variables already included in the model [8]. Models included country-level random effects to account for clustering of respondents within countries and between-country differences. Results are presented as adjusted prevalence ratios (aPRs) with 95% CIs. Interactions between climate concern and age group were also tested, but no evidence of interaction was found. Therefore, these analyses are not presented. As sensitivity analyses, models were re-estimated using a broader threshold for serious climate concern (scores 7–10). Alternative parameterisations were considered, but the distribution was highly skewed towards higher values, and dichotomization provided a more interpretable comparison for this exploratory analysis. Analyses were conducted in Stata17.

## Results

Table S1 presents the sample characteristics. In the EU, 3.1% (95% CI, 2.7–3.6) of participants were current e-cigarette users and 0.9% (0.7–1.2) were current disposable e-cigarette users. Overall, 43.6% (42.6–44.6) considered climate change to be a very serious problem. In the past 6 months, 70.0% (69.1–70.9) reported trying to reduce and separate waste for recycling, and 53.4% (52.4–54.4) reported trying to reduce their use of disposable items whenever possible. Overall, 77.6% (76.8–78.4) reported engaging in at least one of these two pro-environmental behaviours.

**Figure 1.**
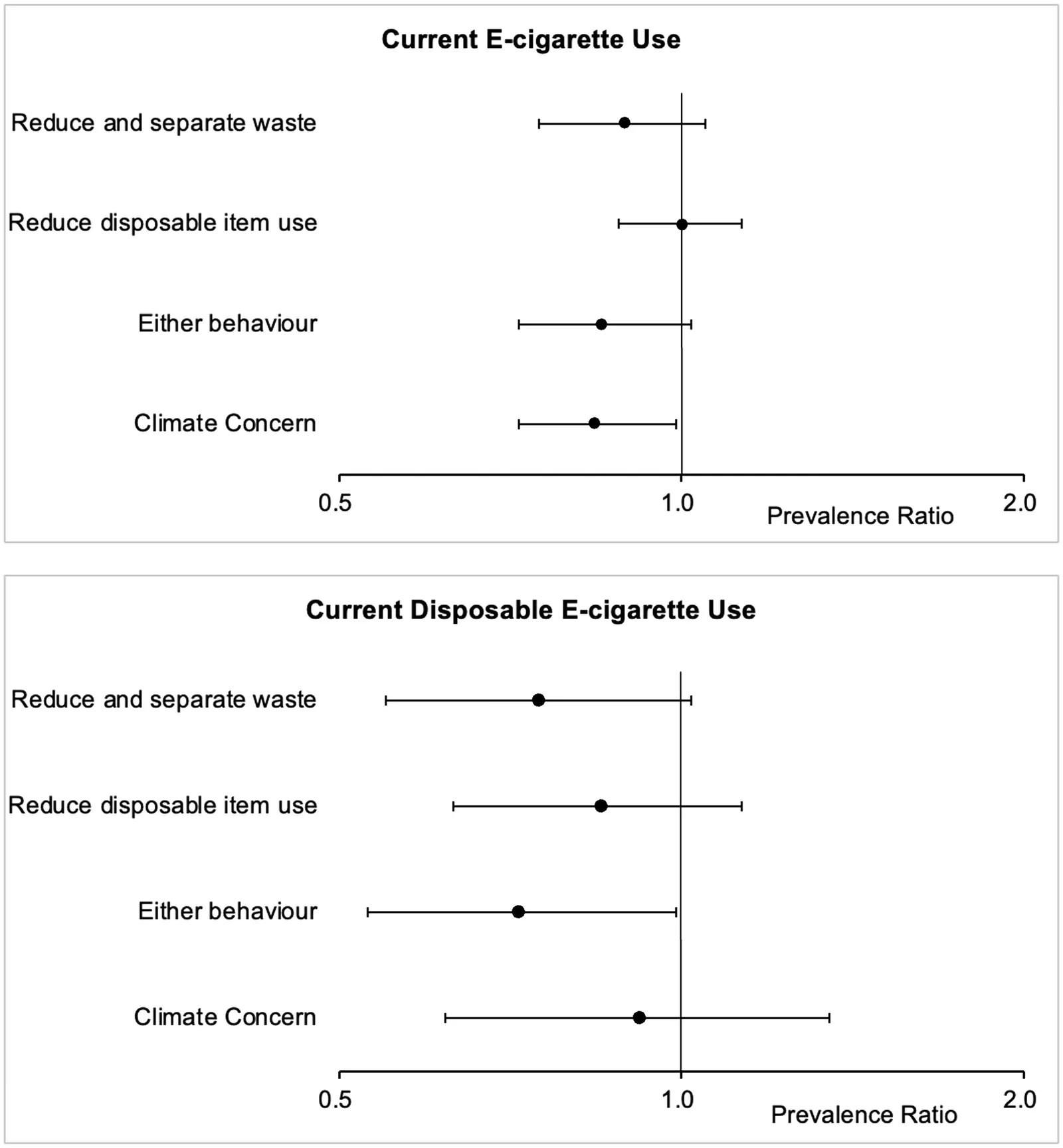
Forest plots of multivariable-adjusted prevalence ratios for the associations of climate concern and pro-environmental behaviours with current e-cigarette use and current disposable e-cigarette use. Note: Multilevel Poisson regression models were adjusted for gender, age, difficulty paying bills, community type, age at completion of full-time education, living with children, and political affiliation. Reduce and separate waste: ‘Which of the following actions, if any, apply to you?’ Response option: You try to reduce your waste and regularly separate it for recycling. Reduce the consumption of disposable items: ‘Which of the following actions, if any, apply to you?’ Response option: You try to cut down on your consumption of disposable items whenever possible (e.g. plastic bags from the supermarket, excess packaging). Either behaviour: Any action involving either waste reduction and separation for recycling or reduced use of disposable items. Climate concern: ‘How serious a problem do you think climate change is at this moment? Please use a scale from 1 to 10, where 1 means not at all a serious problem and 10 means an extremely serious problem’.

After adjustment (Figure 1, Tables S2 and S3), participants who considered climate change to be a very serious problem were less likely to report current e-cigarette use (aPR = 0.84, 95% CI, 0.72–0.99). Those who engaged in at least one of the two pro-environmental behaviours were less likely to report current disposable e-cigarette use (aPR = 0.72, 0.53–0.99), although CIs were wide given the small number of current disposable e-cigarette users. Although the other associations were not statistically significant, several estimates were directionally similar, including for reducing and separating waste for recycling in relation to current disposable e-cigarette use (aPR = 0.75, 0.55–1.02) and for engagement in either pro-environmental behaviour in relation to current e-cigarette use (aPR = 0.85, 0.72–1.02).

## Discussion

Greater climate concern and engagement in pro-environmental behaviours were associated with a lower likelihood of e-cigarette and disposable e-cigarette use, with a clearer pattern for disposable products. While only a subset of associations reached statistical significance, point estimates were directionally consistent across exposure–outcome pairs. Previous EU research has linked health-related perceptions to e-cigarette use [6, 7]; our findings indicate that broader environmental orientations may be similarly relevant.

Individuals with greater climate concern may be less likely to use products perceived as environmentally harmful. E-cigarettes, particularly disposable ones, may increasingly be viewed in this way. The clearer pattern observed for disposable products could relate to increasing policy and public attention to disposable products. Within the EU, bans on disposable e-cigarettes have been introduced in several MS, including Belgium and France, due to their environmental burden and appeal to young people [9]. While concerns regarding electronic, plastic and e-liquid waste may be particularly acute for disposable products, the environmental implications of reusable or refillable e-cigarettes should not be overlooked [2]. Broader environmental orientations may be relevant to e-cigarette use behaviours; however, product-specific environmental perception measures were not assessed in this study and warrant further investigation before informing communication strategies.

At the 11th session of the Conference of the Parties to the WHO Framework Convention on Tobacco Control, the environmental impacts of tobacco and nicotine products and related electronic devices were highlighted [10], reflecting a broader shift in tobacco control towards recognizing environmental damage alongside health harms as an important policy concern. Environmental framing may warrant further investigation in future research on e-cigarette use and regulation.

This study used a large sample from all 27 EU MS and addresses a gap in examining broader environmental orientations and e-cigarette use. However, the cross-sectional design precludes causal inference. Importantly, the absence of product-specific environmental perception measures limits mechanistic interpretation. Furthermore, the climate concern association was sensitive to the chosen threshold, with attenuated associations in sensitivity analyses. The disposable e-cigarette outcome was nested within current e-cigarette use status, which should be considered when comparing findings across the two outcomes; and the composite pro-environmental behaviour measure may have captured individuals with differing behavioural profiles. All variables were self-reported, potentially introducing recall, social desirability, and misclassification bias, while the small number of current disposable e-cigarette users (*n* = 316) could have reduced statistical precision and contributed to wide CIs in some analyses.

In conclusion, this study suggests that climate concern and pro-environmental behaviours may be relevant to e-cigarette use in the EU. These findings should be considered hypothesis-generating, as the measures captured broader environmental orientations rather than direct perceptions of the environmental harms of e-cigarettes. Further research using product-specific environmental measures and direct evaluation of environmental framing approaches is warranted.

## Supplementary Material

ckag114_Supplementary_Data

## Data Availability

The Eurobarometer datasets are owned by the European Commission and can be accessed, either partially or in full, through www.gesis.org. Key points Climate concern and pro-environmental behaviours showed some inverse associations with current e-cigarette and disposable e-cigarette use in the EU. Nearly half of EU respondents reported very high climate concern, and over three-quarters engaged in at least one pro-environmental behaviour. Broader environmental orientations may represent underexplored correlates of e-cigarette use. Broader environmental orientations may warrant further investigation in future research on e-cigarette use, including studies using product-specific environmental perception measures.

## References

[1] European Commission. *Attitudes of Europeans Towards Tobacco and Related Products*. Brussels: European Commission; 2023. https://europa.eu/eurobarometer/surveys/detail/2995 (24 April 2026, date last accessed).

[2] Guraka A , Mierlea S, Drake SJ et al A comprehensive toxicological analysis of panel of unregulated e-cigarettes to human health. Toxicology 2024;509:153964. 39362579 10.1016/j.tox.2024.153964

[3] Ngambo G , Hanna EG, Gannon J et al A scoping review on e-cigarette environmental impacts. Tob Prev Cessat 2023;9:30. 37789930 10.18332/tpc/172079 PMC10542855

[4] European Commission. *Attitudes of Europeans Towards the Environment*. Brussels: European Commission; 2024. https://europa.eu/eurobarometer/surveys/detail/3173 (24 April 2026, date last accessed).

[5] Skeirytė A , Krikštolaitis R, Liobikienė G. The differences of climate change perception, responsibility and climate-friendly behavior among generations and the main determinants of youth’s climate-friendly actions in the EU. J Environ Manage 2022;323:116277. 36137455 10.1016/j.jenvman.2022.116277

[6] Filippidis FT , Laverty AA, Gerovasili V et al Two-year trends and predictors of e-cigarette use in 27 European Union member states. Tob Control 2017;26:98–104. 27220621 10.1136/tobaccocontrol-2015-052771 PMC5256312

[7] Laverty AA , Filippidis FT, Fernandez E et al E-cigarette use and support for banning e-cigarette use in public places in the European Union. Prev Med 2017;105:10–4. 28823683 10.1016/j.ypmed.2017.08.007

[8] Winship C , Radbill L. Sampling weights and regression analysis. Sociol Methods Res 1994;23:230–57.

[9] Directorate for Legal and Administrative Information (Prime Minister). *The Sale of Disposable E-Cigarettes is Now Banned in France* 2025. https://www.service-public.gouv.fr/particuliers/actualites/A18103? lang=en (24 April 2026, date last accessed).

[10] World Health Organization. Implementation of Article 18 of the WHO FCTC. Geneva: World Health Organization, 2025.

