# Supplementary material for: Climate concern, pro-environmental behaviours and use of e-cigarettes in the European Union
Source: Eur J Public Health. 2026 Jun 25;36(4):ckag114. doi: 10.1093/eurpub/ckag114 (PMC13302796; doi:10.1093/eurpub/ckag114)
Supplement: ckag114_Supplementary_Data [file ckag114_supplementary_data.zip › ejph-2026-05-sr-0523-File006.docx]

**Table S4. Sensitivity analysis using a broader climate concern threshold: Multilevel Poisson regression of the associations between climate concern and current e-cigarette and disposable e-cigarette use**

| Variables | Current E–cigarette Use  (*N* = 25,124) | Current Disposable E–cigarette Use  (*N* = 25,120) |
| --- | --- | --- |
|  | Prevalence Ratio (95% Confidence Interval) | |
| Climate concern | | |
| Not serious/Not that serious | 1 | 1 |
| Serious (Score ≥ 7) | 0.86 (0.69-1.08) | 0.74 (0.53-1.04) |
| Gender | | |
| Male (Ref.) | 1 | 1 |
| Female | 0.99 (0.85-1.14) | 1.41 (1.13-1.77) |
| Age (years) | | |
| 55+ (Ref.) | 1 | 1 |
| 15–24 | 7.93 (5.24-12.00) | 19.50 (10.70-35.54) |
| 25–39 | 4.36 (3.23-5.90) | 10.30 (5.39-19.66) |
| 40–54 | 2.25 (1.73-2.94) | 3.75 (2.09-6.72) |
| Difficulty paying bills | | |
| Almost never/never (Ref.) | 1 | 1 |
| From time to time/most of the time | 1.07 (0.87-1.31) | 1.19 (0.87-1.64) |
| Community type | | |
| Rural (Ref.) | 1 | 1 |
| Urban | 1.17 (0.96-1.42) | 1.00 (0.70-1.43) |
| Education (age at completion) | | |
| 0–15 years (Ref.) | 1 | 1 |
| 16–19 years | 1.96 (1.22-3.17) | 1.60 (0.78-3.27) |
| 20+ years | 1.52 (0.93-2.48) | 1.51 (0.71-3.22) |
| Still studying | 1.29 (0.76-2.20) | 1.49 (0.65-3.39) |
| Living with children | | |
| No (Ref.) | 1 | 1 |
| Yes | 1.11 (0.86-1.41) | 1.11 (0.70-1.76) |
| Political affiliation | | |
| Centre (Ref.) | 1 | 1 |
| Left | 1.27 (1.01-1.59) | 1.30 (0.96-1.77) |
| Right | 1.10 (0.85-1.42) | 1.21 (0.82-1.78) |
| Don't know/Didn't respond | 0.94 (0.75-1.18) | 0.86 (0.56-1.33) |

**Note:**

Climate concern: ‘How serious a problem do you think climate change is at this moment? Please use a scale from 1 to 10, where 1 means not at all a serious problem and 10 means an extremely serious problem.’
