# Supplementary material for: Climate concern, pro-environmental behaviours and use of e-cigarettes in the European Union
Source: Eur J Public Health. 2026 Jun 25;36(4):ckag114. doi: 10.1093/eurpub/ckag114 (PMC13302796; doi:10.1093/eurpub/ckag114)
Supplement: ckag114_Supplementary_Data [file ckag114_supplementary_data.zip › ejph-2026-05-sr-0523-File003.docx]

**Table S1. Sample characteristics**

| Variables | *N* | Weighted Proportion (%) with 95% Confidence Interval |
| --- | --- | --- |
| **Current e–cigarette use** | | |
| Never/former user | 25,193 | 96.9% (96.4–97.3) |
| Current user | 766 | 3.1% (2.7–3.6) |
| **Current disposable e–cigarette use** | | |
| Never/former user | 25,638 | 99.1% (98.8–99.3) |
| Current user | 316 | 0.9% (0.7–1.2) |
| **Climate concern** | | |
| Less than very serious | 15,709 | 56.4% (55.4–57.4) |
| Very serious (Score ≥ 9) | 10,499 | 43.6% (42.6–44.6) |
| **Climate concern (sensitivity analysis)** | | |
| Not serious/Not that serious | 6,767 | 22.9% (22.1–23.7) |
| Serious (Score ≥ 7) | 19,441 | 77.1% (76.3–77.9) |
| **Reduce and separate waste** | | |
| Yes | 18,020 | 70.0% (69.1–70.9) |
| **Reduce the consumption of disposable items** | | |
| Yes | 14,515 | 53.4% (52.4–54.4) |
| **Waste reduction/separation or fewer disposable items** | | |
| Yes | 20,279 | 77.6% (76.8–78.4) |
| **Gender** | | |
| Man | 12,179 | 48.5% (47.5–49.5) |
| Woman | 14,122 | 51.5% (50.5–52.5) |
| **Age (years)** | | |
| 15–24 | 2,385 | 12.3% (11.6–13.1) |
| 25–39 | 5,168 | 22.0% (21.2–22.9) |
| 40–54 | 6,575 | 25.1% (24.2–25.9) |
| 55+ | 12,222 | 40.6% (39.6–41.5) |
| **Difficulty paying bills** | | |
| Almost never/never | 17,325 | 65.8% (64.8–66.7) |
| From time to time/most of the time | 8,845 | 34.2% (33.3–35.2) |
| **Community type** | | |
| Rural | 8,971 | 31.4% (30.5–32.3) |
| Urban | 17,373 | 68.6% (67.7–69.5) |
| **Education (age at completion)** | | |
| 0–15 years | 2,968 | 13.5% (12.9–14.3) |
| 16–19 years | 11,187 | 43.5% (42.5–44.5) |
| 20+ years | 9,856 | 33.3% (32.3–34.2) |
| Still studying | 1,971 | 9.7% (9.1–10.4) |
| **Living with children** | | |
| Yes | 8,620 | 32.9% (31.9–33.8) |
| **Political affiliation** | | |
| Centre | 14,881 | 59.2% (58.2–60.1) |
| Left | 4,138 | 16.2% (15.5–17.0) |
| Right | 3,597 | 11.8% (11.2–12.4) |
| Don't know/Didn't respond | 3,737 | 12.8% (12.2–13.5) |
| ***N*** | 26,353 |  |

**Note:**

Subcategory counts may not sum to the total *N* due to missing values on individual variables.

Climate concern: ‘How serious a problem do you think climate change is at this moment? Please use a scale from 1 to 10, where 1 means not at all a serious problem and 10 means an extremely serious problem.’

Reduce and separate waste: ‘Which of the following actions, if any, apply to you?’ Response option: You try to reduce your waste and regularly separate it for recycling.

Reduce the consumption of disposable items: ‘Which of the following actions, if any, apply to you?’ Response option: You try to cut down on your consumption of disposable items whenever possible (e.g. plastic bags from the supermarket, excess packaging).

Waste reduction/separation or fewer disposable items: Any action involving either of the two behaviours listed above.
