# Supplementary material for: Climate concern, pro-environmental behaviours and use of e-cigarettes in the European Union
Source: Eur J Public Health. 2026 Jun 25;36(4):ckag114. doi: 10.1093/eurpub/ckag114 (PMC13302796; doi:10.1093/eurpub/ckag114)
Supplement: ckag114_Supplementary_Data [file ckag114_supplementary_data.zip › ejph-2026-05-sr-0523-File005.docx]

**Table S3. Multilevel Poisson regression of the associations between climate concern, pro–environmental behaviours, and disposable e–cigarette use**

| Variables | Current Disposable E–cigarette Use    Prevalence Ratio (95% Confidence Interval) | | | | | | |
| --- | --- | --- | --- | --- | --- | --- | --- |
|  | Climate concern  (*N* = 25,120) | | Reduce and separate waste  (*N* = 25,139) | | Reduce disposable item use  *(N* = 25,139) | | Either behaviour  *(N* = 25,139) |
| Climate concern | | | | | | | |
| Less than very serious | 1 | – | | – | | – | |
| Very serious (Score ≥ 9) | 0.92 (0.62–1.35) | – | | – | | – | |
| Reduce and separate waste | | | | | | | |
| No (Ref.) | – | 1 | | – | | – | |
| Yes | – | 0.75 (0.55–1.02) | | – | | – | |
| Reduce disposable item use | | | | | | | |
| No (Ref.) | – | – | | 1 | | – | |
| Yes | – | – | | 0.85 (0.63–1.13) | | – | |
| Waste reduction/separation or fewer disposable items | | | | | | | |
| No (Ref.) | – | – | |  | | 1 | |
| Yes | – | – | |  | | 0.72 (0.53–0.99) | |
| Gender | | | | | | | |
| Male (Ref.) | 1 | 1 | | 1 | | 1 | |
| Female | 1.38 (1.10–1.74) | 1.39 (1.10–1.75) | | 1.39 (1.10–1.75) | | 1.40 (1.10–1.76) | |
| Age (years) | | | | | | | |
| 55+ (Ref.) | 1 | 1 | | 1 | | 1 | |
| 15–24 | 19.09 (10.39–35.09) | 18.22 (9.70–34.21) | | 18.70 (10.16–34.42) | | 18.46 (9.97–34.19) | |
| 25–39 | 10.25 (5.35–19.62) | 10.02 (5.14–19.51) | | 10.24 (5.33–19.66) | | 10.10 (5.24–19.47) | |
| 40–54 | 3.75 (2.09–6.73) | 3.71 (2.06–6.69) | | 3.76 (2.10–6.74) | | 3.73 (2.08–6.68) | |
| Difficulty paying bills | | | | | | | |
| Almost never/never (Ref.) | 1 | 1 | | 1 | | 1 | |
| From time to time/most of the time | 1.20 (0.87–1.67) | 1.18 (0.84–1.66) | | 1.19 (0.85–1.67) | | 1.18 (0.84–1.64) | |
| Community type | | | | | | | |
| Rural (Ref.) | 1 | 1 | | 1 | | 1 | |
| Urban | 1.00 (0.70–1.42) | 1.01 (0.71–1.43) | | 1.01 (0.71–1.44) | | 1.01 (0.71–1.44) | |
| Education (age at completion) | | | | | | | |
| 0–15 years (Ref.) | 1 | 1 | | 1 | | 1 | |
| 16–19 years | 1.59 (0.77–3.27) | 1.63 (0.80–3.33) | | 1.60 (0.78–3.28) | | 1.62 (0.80–3.30) | |
| 20+ years | 1.48 (0.69–3.18) | 1.52 (0.71–3.25) | | 1.49 (0.70–3.18) | | 1.51 (0.71–3.19) | |
| Still studying | 1.46 (0.64–3.33) | 1.53 (0.66–3.52) | | 1.48 (0.66–3.36) | | 1.51 (0.67–3.42) | |
| Living with children | | | | | | | |
| No (Ref.) | 1 | 1 | | 1 | | 1 | |
| Yes | 1.10 (0.70–1.74) | 1.11 (0.71–1.76) | | 1.11 (0.71–1.74) | | 1.11 (0.71–1.74) | |
| Political affiliation | | | | | | | |
| Centre (Ref.) | 1 | 1 | | 1 | | 1 | |
| Left | 1.29 (0.94–1.77) | 1.29 (0.95–1.75) | | 1.29 (0.95–1.76) | | 1.30 (0.96–1.76) | |
| Right | 1.21 (0.83–1.77) | 1.19 (0.83–1.71) | | 1.19 (0.83–1.71) | | 1.18 (0.83–1.70) | |
| Don't know/Didn't respond | 0.87 (0.57–1.32) | 0.87 (0.57–1.31) | | 0.87 (0.57–1.31) | | 0.87 (0.57–1.31) | |

**Note:**

Dashes indicate that the variable was not included in that model.

Climate concern: ‘How serious a problem do you think climate change is at this moment? Please use a scale from 1 to 10, where 1 means not at all a serious problem and 10 means an extremely serious problem.’

Reduce and separate waste: ‘Which of the following actions, if any, apply to you?’ Response option: You try to reduce your waste and regularly separate it for recycling.

Reduce the consumption of disposable items: ‘Which of the following actions, if any, apply to you?’ Response option: You try to cut down on your consumption of disposable items whenever possible (e.g. plastic bags from the supermarket, excess packaging).

Waste reduction/separation or fewer disposable items: Any action involving either of the two behaviours listed above.
