# Supplementary material for: Climate concern, pro-environmental behaviours and use of e-cigarettes in the European Union
Source: Eur J Public Health. 2026 Jun 25;36(4):ckag114. doi: 10.1093/eurpub/ckag114 (PMC13302796; doi:10.1093/eurpub/ckag114)
Supplement: ckag114_Supplementary_Data [file ckag114_supplementary_data.zip › ejph-2026-05-sr-0523-File004.docx]

**Table S2. Multilevel Poisson regression of the associations between climate concern, pro–environmental behaviours, and e–cigarette use**

| Variables | Current E–cigarette Use    Prevalence Ratio (95% Confidence Interval) | | | | | | |
| --- | --- | --- | --- | --- | --- | --- | --- |
|  | Climate concern  (*N* = 25,124) | | Reduce and separate waste  (*N* = 25,143) | | Reduce disposable item use  *(N* = 25,143) | | Either behaviour  *(N* = 25,143) |
| Climate concern | | | | | | | |
| Less than very serious | 1 | – | | – | | – | |
| Very serious (Score ≥ 9) | 0.84 (0.72–0.99) | – | | – | | – | |
| Reduce and separate waste | | | | | | | |
| No (Ref.) | – | 1 | | – | | – | |
| Yes | – | 0.89 (0.75–1.05) | | – | | – | |
| Reduce disposable item use | | | | | | | |
| No (Ref.) | – | – | | 1 | | – | |
| Yes | – | – | | 1.00 (0.88–1.13) | | – | |
| Waste reduction/separation or fewer disposable items | | | | | | | |
| No (Ref.) | – | – | | – | | 1 | |
| Yes | – | – | | – | | 0.85 (0.72–1.02) | |
| Gender | | | | | | | |
| Male (Ref.) | 1 | 1 | | 1 | | 1 | |
| Female | 0.99 (0.85–1.15) | 0.98 (0.84–1.15) | | 0.98 (0.84–1.13) | | 0.98 (0.85–1.15) | |
| Age (years) | | | | | | | |
| 55+ (Ref.) | 1 | 1 | | 1 | | 1 | |
| 15–24 | 7.86 (5.19–11.89) | 7.70 (5.11–11.60) | | 7.80 (5.18–11.73) | | 7.72 (5.13–11.60) | |
| 25–39 | 4.36 (3.23–5.88) | 4.32 (3.18–5.85) | | 4.35 (3.22–5.89) | | 4.33 (3.20–5.84) | |
| 40–54 | 2.25 (1.73–2.94) | 2.26 (1.73–2.95) | | 2.27 (1.74–2.96) | | 2.26 (1.73–2.95) | |
| Difficulty paying bills | | | | | | | |
| Almost never/never (Ref.) | 1 | 1 | | 1 | | 1 | |
| From time to time/most of the time | 1.07 (0.88–1.31) | 1.06 (0.87–1.31) | | 1.07 (0.88–1.31) | | 1.06 (0.87–1.30) | |
| Community type | | | | | | | |
| Rural (Ref.) | 1 | 1 | | 1 | | 1 | |
| Urban | 1.17 (0.96–1.42) | 1.18 (0.97–1.42) | | 1.18 (0.98–1.42) | | 1.18 (0.98–1.42) | |
| Education (age at completion) | | | | | | | |
| 0–15 years (Ref.) | 1 | 1 | | 1 | | 1 | |
| 16–19 years | 1.96 (1.22–3.16) | 1.99 (1.20–3.27) | | 1.97 (1.20–3.24) | | 1.98 (1.21–3.27) | |
| 20+ years | 1.52 (0.94–2.48) | 1.54 (0.94–2.52) | | 1.52 (0.94–2.46) | | 1.53 (0.94–2.50) | |
| Still studying | 1.30 (0.77–2.20) | 1.31 (0.75–2.28) | | 1.29 (0.75–2.22) | | 1.31 (0.76–2.26) | |
| Living with children | | | | | | | |
| No (Ref.) | 1 | 1 | | 1 | | 1 | |
| Yes | 1.10 (0.86–1.41) | 1.11 (0.86–1.43) | | 1.10 (0.86–1.42) | | 1.11 (0.86–1.42) | |
| Political affiliation | | | | | | | |
| Centre (Ref.) | 1 | 1 | | 1 | | 1 | |
| Left | 1.28 (1.02–1.61) | 1.25 (1.00*–1.57) | | 1.25 (0.99–1.57) | | 1.25 (1.00*–1.57) | |
| Right | 1.11 (0.86–1.43) | 1.09 (0.85–1.41) | | 1.10 (0.85–1.41) | | 1.09 (0.85–1.40) | |
| Don't know/Didn't respond | 0.94 (0.75–1.18) | 0.95 (0.76–1.20) | | 0.95 (0.76–1.20) | | 0.95 (0.76–1.20) | |

**Note:**

Dashes indicate that the variable was not included in that model.

* Indicates that the unrounded confidence interval limit is below 1.00.

Climate concern: ‘How serious a problem do you think climate change is at this moment? Please use a scale from 1 to 10, where 1 means not at all a serious problem and 10 means an extremely serious problem.’

Reduce and separate waste: ‘Which of the following actions, if any, apply to you?’ Response option: You try to reduce your waste and regularly separate it for recycling.

Reduce the consumption of disposable items: ‘Which of the following actions, if any, apply to you?’ Response option: You try to cut down on your consumption of disposable items whenever possible (e.g. plastic bags from the supermarket, excess packaging).

Waste reduction/separation or fewer disposable items: Any action involving either of the two behaviours listed above.
